# Characterization of Seed Storage Proteins from Chickpea Using 2D Electrophoresis Coupled with Mass Spectrometry

**DOI:** 10.1155/2016/1049462

**Published:** 2016-04-10

**Authors:** Pramod Kumar Singh, Nidhi Shrivastava, Krishna Chaturvedi, Bechan Sharma, Sameer S. Bhagyawant

**Affiliations:** ^1^School of Studies in Biotechnology, Jiwaji University, Gwalior 474011, India; ^2^Department of Bioscience & Biotechnology, Banasthali University, Banasthali 304022, India; ^3^Defense Research & Development Establishment, Gwalior 474011, India; ^4^Department of Biochemistry, University of Allahabad, Allahabad 211002, India

## Abstract

Proteomic analysis was employed to map the seed storage protein network in landrace and cultivated chickpea accessions. Protein extracts were separated by two-dimensional gel electrophoresis (2D-GE) across a broad range 3.0–10.0 immobilized pH gradient (IPG) strips. Comparative elucidation of differentially expressed proteins between two diverse geographically originated chickpea accessions was carried out using 2D-GE coupled with mass spectrometry. A total of 600 protein spots were detected in these accessions. In-gel protein expression patterns revealed three protein spots as upregulated and three other as downregulated. Using trypsin in-gel digestion, these differentially expressed proteins were identified by matrix-assisted laser desorption ionization time of flight mass spectrometry (MALDI-TOF-MS) which showed 45% amino acid homology of chickpea seed storage proteins with* Arabidopsis thaliana*.

## 1. Introduction

The postgenomic era is acknowledged for proteomics as a next frontier for biological research. Currently, extensive information related to biological functions is being created through proteomic research interlocked with genomic research. In plants, only few species have available complete genomic information. In this context, proteomics technology has provided unique opportunities to develop strategies for exploring seed biology research for human benefit. Comparative or quantitative proteomics is the principally utilized subarea of proteomics [1, 2]. It aims at ascertaining the differences in protein profiles between two samples from different individuals or from distinct treatments. Proteomics technology provides a high throughput technique for cultivar authentication in many crops. Versatility of 2D-GE coupled with mass spectroscopy investigates and reveals hundreds of proteins simultaneously to their different isoforms and posttranslational modifications which may act as marker for cultivar identification. In recent years, proteomic characterization has been employed in some major crops like wheat [3], rice [4], barley [5], lupin [6], soybean [7], and mungbean [8] whose genomes have been fully or extensively characterized.

Legumes are known for their nutritive values that play an important role in human nutrition and serve as supplement to improve growth of livestock [9, 10]. Chickpea (*Cicer arietinum* L.) is the third most important legume crop in the world (http://www.fao.org/). In India, chickpea seeds are the preferred source of protein since they have a rich source of digestible proteins. Seed storage proteins (SSP) of chickpea are mainly comprised of albumin, globulin, and glutelin [11]. Genetic variability within the germplasm pool can be utilized to identify elite chickpea accessions of agronomic importance. Landrace chickpea accessions are more diverse than cultivated chickpea. Some of such landrace accessions may contain proteins with properties better than those of cultivated varieties, which can further be used for chickpea improvement [7, 12]. Therefore, a comparative analysis using proteomic tools of seed proteins from various genotypes including landrace and cultivated is important for better understanding of wide range of seed proteins. The present attempt describes the comparative profiling of chickpea seed storage proteins in landrace and cultivated chickpea accessions using 2D-GE coupled with mass spectroscopy which may assist in understanding attributes related to their functional characteristics.

## 2. Experimental Design

### 2.1. Plant Material

The study was carried out with landrace and cultivated chickpea accessions. The landrace chickpea accession ICC11284 has multiple disease resistance features and originates from Union of Soviet Socialist Republic (USSR). The seeds of this landrace accession were collected from International Crop Research Institute for Semi-Arid Tropics (ICRISAT), Patancheru, Hyderabad (AP) India. The Indian origin cultivated chickpea var. JAKI9218 with early maturity and high yield and resistant to* Fusarium* was procured from Agricultural College, Sehore (MP), India.

### 2.2. Chickpea Total Protein Extract Preparation

Total seed storage proteins were extracted using the procedure [6]. The defatted chickpea flour was extracted with a solution consisting of 7 M urea, 2 M thiourea, 2% CHAPS, and 65 mM 1,4-dithiothreitol (DTT) in a ratio of 1/30 (w/v) under stirring at room temperature for 2 h. The slurry was centrifuged at 10,000 ×g for 30 min at 4°C and the extracted proteins in the supernatant were analyzed immediately.

### 2.3. 2D-GE Analysis

The extracted proteins were subjected to 2D-GE analysis as per the standard procedure [13]. The isoelectric focusing (IEF) was performed using 7 cm, pH 3–10 gradient IPG strips (Bio-Rad, USA). The strips were rehydrated overnight in a solution containing 7 M urea, 2% w/v CHAPS, 15 mM DTT, and 0.5% v/v IPG buffer pH 3–10 (Bio-Rad, USA) containing the protein sample. For the first dimension, 300 *μ*g of protein sample was loaded. These amounts were optimized for the best electrophoretic performance. After 16 h of passive rehydration at 20°C, isoelectric focusing was performed and strips were focused initially at 250 V for 3 h till 8000 volt hours under mineral oil. Strips of IPG were equilibrated for total 25 min prior to SDS-PAGE. After the first dimension, strips were equilibrated for 15 min in the equilibration buffer-I (50 mM Tris-HCl buffer, pH 8.8 containing 6 M urea, 30% w/v glycerol, 2% SDS, and 1% DTT) and then for 10 min in the equilibration buffer-II (equilibration buffer I containing 4% w/v iodoacetamide instead of DTT). After equilibration, strips were transferred to 12% SDS-PAGE for second-dimension separation at a constant voltage of 200 V for 3 h. Following electrophoresis, 2D gels were visualized by staining with Colloidal Coomassie Blue G-250. Protein spots were visualized under white light in a UV transilluminator at 280 nm.

### 2.4. In-Gel Enzymatic Digestion of Protein and MALDI-TOF Analysis

Selected protein spots were excised from the 2D-GE gel by modified Gilson pipette tips, transferred to sterilized 0.5 mL tubes and stored in 50% ethanol until digestion. After removing ethanol solution, gel pieces were incubated in 100 *μ*L distilled water for 15 min at room temperature and then in 40 *μ*L 50% acetonitrile (ACN) for the same time. This step was repeated three times. Subsequently the supernatants were removed and excised gel fragments were equilibrated with 40 *μ*L 50% ACN containing 25 mM ammonium bicarbonate to remove Coomassie Blue stain. After removing the supernatants, gel pieces were dehydrated with 40 *μ*L 100% ACN and dried under vacuum on a centrifugal evaporator. For protein digestion, 20 *μ*L of 10 *μ*g/mL trypsin in 25 mM ammonium bicarbonate containing 2.5 mM CaCl_2_, pH 7.8, was added to each sample and incubated overnight at 37°C. The resulting tryptic fragments were extracted from the gel with 50% ACN and 5% trifluoroacetic acid (TFA) by sonication. The extract was dried to completeness and dissolved in 50% ACN and 0.1% TFA. For mass spectrometry analysis, the resulting peptide mixtures were combined with the matrix solution containing 10 mg/mL of cyanohydroxycinnamic acid (CHCA) in 50% ACN/0.1% TFA and applied to sample plate prior to analysis by MALDI-TOF-MS. Instruments used for analysis were 4800 MALDI TOF/TOF Analyzer (Applied Biosystems), QStar Elite coupled to Tempo Nano MDLC (Applied Biosystems) equipped with ion spray source running analyst QS software, and ultimate 3000 Nano HPLC system (Dionex) coupled to a 4000 QTRAP mass spectrometer (Applied Biosystems). Acquisitions were performed in data-dependent MS/MS scanning mode (full MS scan range of 400–2000* m/z*). Proteins were identified on the basis of their matches to proteins in other species using MASCOT search engine (http://www.matrixscience.com/).

## 3. Results and Discussion

**Figure 1:**
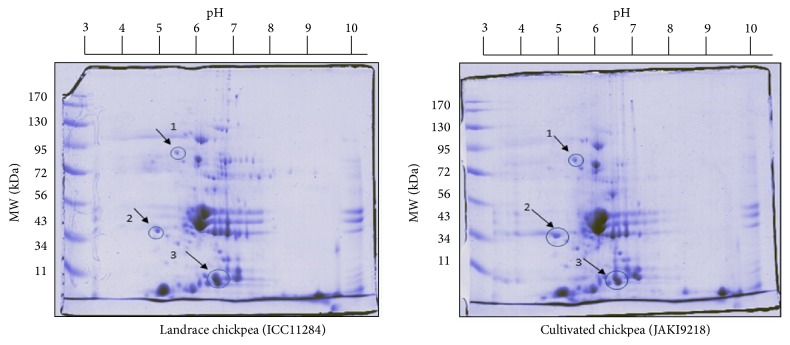
2D gel fingerprint pattern of landrace and cultivated chickpea accessions using 3.0–10.0 IPG strips.

The present study involved 2D-GE coupled with mass spectrometry to analyze the posttranslational expressions of seed storage proteins in landrace and cultivated chickpea accessions. The proteins were resolved into 7 cm IPG strip having a pH gradient of 3–10 and subsequently separated on the basis of mass using SDS-PAGE in second dimension.  Figure 1 shows 2D-GE profile of both accessions in a molecular weight range of 11–170 kDa. 2D-GE protein fingerprint map reveals intrinsically polymorphic pattern of* C. arietinum* L. storage proteins with several spots of same molecular weights. Different pIs point towards charge heterogeneity in both landrace and cultivated chickpea accessions. Six clear differentially expressed individual protein spots were identified and manually excised, trypsin digested, and processed for downstream MALDI-TOF-MS analysis. The majority of proteins showed apparent molecular masses in the range of 10–80 kDa for both accessions. Proteins samples were subjected to 2D-GE in duplicate with the same conditions to observe consistency in expression patterns. The experimental masses and pIs were consistent to reproduce and differentially expressed proteins were selected for mass spectrometry analysis.

**Figure 2:**
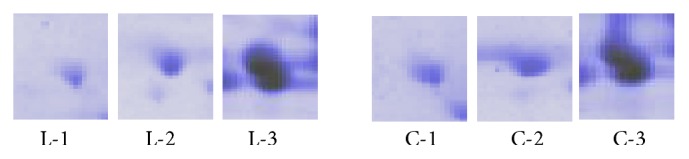
Magnified view showing differentially expressed chickpea protein spots in landrace (L) and cultivated (C) chickpea accessions.

Finally, all the selected protein spots were subjected to mass spectrometry analysis and for candidate protein identification using MASCOT (http://www.matrixscience.com/) search engine. Magnified views of some protein spots showed clear differential expression between landrace and cultivated chickpea accessions as shown in Figure 2. The query search of these amino acid sequences found significant correlations with legumin *α* and *β* subunit precursor of* C. arietinum* L. Mass spectrometry data showed 45% amino acid homology of chickpea seed storage proteins with* Arabidopsis thaliana*. Some protein spots were assigned through the identification of homologous gene products from other seeds like* Oryza sativa* allowing only relatively low amino acid coverage values (Table 1). The proteomic tools have recently been employed to test and detect allergen, antinutritional proteins, and elicitors of disease resistance in many seed genotypes. Sea weed (*Hypnea musciformis*) polysaccharides act as an elicitor of disease resistance responses in chickpea (*C. arietinum* L). Fatima et al. [14] reported components of the induced phytoalexin, that is, isoflavonoids and their glycoconjugates, using LC-MS techniques. Seed storage proteins and seed maturation proteins are synthesized during the later stages of seed development. These maturation proteins are different from late embryogenesis abundant proteins (LEA proteins) that commonly accumulate to high levels during the late stages of seed maturation. The major features of LEA proteins are low sequence complexity; occurrence of repeat motifs; heat solubility; and an apparent lack of defined structure.

Present analysis showed total of six differentially expressed seed storage proteins in chickpea by 2D-GE analysis. Earlier study by Pandey et al. [15] while comparing seed storage proteins reported approximately 600 protein spots in chickpea on 2D-GE and compared them with the proteomes of* Arabidopsis* and rice. These workers reported only eight identical proteins in all the three organisms. According to these workers, 71% of the chickpea nuclear proteins are novel which suggest further investigation for a better understanding of the nuclear proteome. In another study, Magni et al. [6] have introduced combined 2D electrophoretic approaches for study of white lupin seed storage proteins useful for fundamental investigations. In their extended studies they identified complex regulatory network responses of nuclear proteins against dehydration stress in chickpea. These reports extend platform and basis to undertake future investigations of nuclear proteins networks in chickpea and other legumes. A number of researchers have pointed out that the 2D-GE data can be employed in the context of climatic changes [16] vis-à-vis osmotic stress including drought, salt, flooding, and metal stress. Flooding stress leads to shifting alternative pathways of energy generation. Schneider et al. [17] investigated the vacuolar proteome of mesophyll cells of barley leaves in response to cadmium stress. However, such studies in chickpea are scanty which throw light on tracking essential stress responsible pathways. The present study explored differentially expressed low abundance protein and/or seed storage proteins in chickpea which extend information on the protein networks and might help in better understanding regarding their expression patterns at maturity level. However, to strengthen further, use of second-generation proteomics technologies such as iTRAQ peptide tagging system may be employed to investigate the responses of crop plants against a variety of agricultural hindrance/stresses.

Proteomics put forward a powerful tool to study the alteration of protein levels against plant defense mechanism. Peptide mass fingerprinting (PMF) acquired by MALDI-TOF-MS remains the most sophisticated and powerful techniques of protein identification. This approach can successfully be applied and is more efficient for those plant species whose genomes have already been sequenced and fully annotated. However, for species without full genome sequence, when ESTs are available, it is still possible to carry out identifications using such strategy. Species-specific EST databases have been used for protein identification as an alternative in plant species without full genome sequence information [18–22]. The present comparative proteome analysis of landrace and cultivated chickpea accessions profiles analyzed by 2D-GE revealed proteins which were differentially expressed. Among them, three spots were upregulated and three protein spots were downregulated. Mascot query search revealed 35% of conserved homology with sequences of* M. truncatula* and 17% with* legumin* *α* and *β* subunit precursors of* Cicer arietinum* L. in a pI range of 6.20. The other excised chickpea proteins exhibited association with species of* Vicia narbonensis* at insignificant levels. However, chickpea seed storage proteins showed maximum linkages with* Arabidopsis thaliana* of 45%. As such, the present investigation provides useful protein homology information of landrace as well as cultivated accessions of chickpea and these seed storage protein profiles could practically be useful biomarkers in the studies of genetic diversity.

## Figures and Tables

**Table 1:** Identification of mature subunits in proteolytically processed proteins of chickpea.

Spot number	Full length deduced amino acid sequence	Subunit identification	MW	pI	Amino acid CoV, %

1	MEE­IVK­RFL­GSE­CFS­STF­IK**E**­**FWD­VMK­WK**V**­**LSR­RLA­EVI­GTK­NTF­CIH­K**RE**­**VLM­DGV­FVI­NGV­NDI­AKR­**LDK­KRL­DSK­GGL	*Medicago truncatula*	**9517**	**9.51**	35

2	M**AK**­**LLA­LSL­SFC­FLL­FGT­CFA­LR**D**­**QPQ­QNE­CQL­EHL­NAL­KPD­NRI­KSE­GGL­IET­WNP­SNK­QFA­CAG­VAL­SRA­TLQ­PNS­LLQ­TFL­HQR­SPE­IFI­QQG­NGY­FGM­VFP­GCV­ETF­EEP­RES­EQG­EGS­KFS­DSH­QKV­NRF­REG­DII­AVP­TGV­VFW­MFN­DQD­TPV­IAV­SLI­DTS­SFQ­NQL­DQM­PRR­FYL­AGN­HEQ­EFL­R**YQ**­**QEG­SEE­EEN­EGG­NIF­SGF­K**R**D­FLE­DAL­NVN­R**RI**­**VNK­LQG­RNE­DEE­KGA­IVK­VKG­GLS­ITT­PPE­KEP­RQK­RGS­RQE­EDE­DED­EKR­QPH­RHS­RQD­EDE­DEK­RQP­HHH­SRG­GSK­SQR­DNG­FEE­TIC­TAR­**LHQ­NIG­SSS­SPD­IYN­PQA­GR**I**­**KTV­TSF­DLQ­ALR­FLK­LSA­EFG­SLH­KNA­MFV­PHY­NLN­ANS­ILY­ALK­GRA­RLL­YAL­NCK­**GNS­VFD­GEL­EAG­R**AL**­**IVP­QNF­AIA­AKS­LSD­RFS­YVA­FKT­NDR­ALI­NVC­QKK­LLQ­LLS­IWK­EMR­PGS­SSS­TAP­FHF­LFH­PAV­TQT­TKQ­QLD­LVP­NQY­E	*Legumin*, alpha and beta, subunit precursor of *Cicer arietinum*	**56672**	**6.20**	**17**

3	MAK­LLA­LSL­SLC­FLL­FSN­SFA­LRE­QSQ­QNE­CQL­ERL­DAL­EPD­NRI­ESE­GGL­IET­WNP­NNR­QFR­CAG­VAL­SRV­TLQ­RNA­LRR­PYY­SNA­PQE­IYI­QQG­NGY­FGV­VFP­GCP­ETF­EEP­QES­EQR­ERR­RYR­DSH­QKV­NRF­REG­DII­AVP­TGN­VLW­MYN­DQD­TPV­IAI­SLT­DTG­SSN­NQL­DQI­PRR­FYL­AGN­QEQ­EFL­RYQ­REQ­GKQ­EQE­NDG­NNI­FSG­FKR­**DFL­EDA­LNV­NR**H**­**IVD­RLQ­GRN­EDE­EKG­AIV­KVK­GGL­SII­TPP­ERQ­RGS­RQE­EDE­DEK­EER­QPS­RRR­DES­QKG­ESR­RHG­DNG­LEE­TVC­TAK­**LRV­NIG­SSP­SPD­IYN­PQA­GR**I**­**NTV­TSL­DLP­VLR­WLK­LSA­EHG­SLR­KNA­LIV­PHY­NR**N**­**ANS­VIY­ALK­GR**A**­**RLQ­VVN­CNG­NTV­FDG­ELE­AGR­ALT­VPQ­NYA­VAA­KSL­SER­FTY­VAF­KTN­DRD­GIA­RLA­GTS­SVI­NDL­PLD­VVA­ATF­NLQ­RNE­ARQ­LKS­NNP­FKL­LVP­PRE­SEK­RAS­A	*Vicia narbonensis*	**54977**	**7.00**	8

4	MDE­VVA­TMD­ISE­ANE­GYA­SCG­SVI­EMS­RQM­KTT­RVG­ARA­QPW­PAC­PGI­PAV­GRV­GSV­LLF­TAR­**MGE­GLC­HMF­TTG­SK**A**­**MEC­GVK­MCL­VGW­PGL­AWD­ELG­RSG­CQF­GLN­HRR­PWV­KAV­LDG­QVS­EEE­DIV­SCL­PKL­QKT­AGS­ASE­VEE­AVK­PAV­KQD­KRL­RSV­KVL­SLV­SNL­SLP­FVF­PLS­LSK­PLQ­QMA­DNE­KGN­KVR­SQD­IGT­SSS­RVN­EAP­DTS­CVA­VVQ­HLI­NQN­KLL­IEI­LQQ­HRM­PIL­NPS­QMQ­PQV­QLE­AVQ­ALT­PQV­SAP­PTS­QKA­PHA­MPH­VDP­KEA­SII­CFM­CDE­QGH­YAR­NCP­QQK­RK**A**­**PMR­TED­EVR­**KMI­ITS­TEW­PPP­GMT­KHQ­RRN­SFR­GAQ­QLT­EHL­LAN­GGR­VSG­SED­SDQ­VSS­DDE­DEK­SPQ­GFN­QNK­IEL­KAC­SRC­GEI­GHV­ASS­CAS­TCV­HCE­EDH­PPD­RCP­TNK­ITY­FFC­EGT­DHV­PKD­CQF­SFL­LTK­KMA­NQP­ASS­NGE­KHQ­GNT­NPR­QDH­RFS­LTP­VPG­QRN­RNE­KRK­CRV­RED­ICC­FNC­QGM­GHF­ADK­CPK­PRN­IAA­GTS­VHA­TPC­NQK­LAP­QRI­VIH­ASR­SSP­IAR­VAT­API­PMN­ALP­QGV­NAQ­FQP­QPP­ADK­TGA­SIC­VVP­LEV­PIQ­QLR­NQV­QDE­EPE­CKK­VIV­CYN­CSE­EGH­YSK­NCP­QPR­QNR­PPH­YRQ­FTR­SRH­SNR­IVV­TGA­NAV­PVR­PRV­NQN­P	*Oryza sativa* (rice)	**73339**	**8.75**	**3**

5	MAK­LLA­LSL­SFC­FLL­FGT­CFA­LRD­QPQ­QNE­QLE­HLN­ALK­PDN­RIK­SEG­GLI­ETW­NPS­NKQ­FAC­AGV­ALS­RAT­LQP­NSL­LQT­FLH­QRS­PEF­IQQ­GNG­YFG­MVF­PGC­VET­FEE­PRE­SEQ­GGS­KFS­DSH­QKV­NRF­REG­DII­AVP­TGV­VFW­MFN­DQD­TPV­IAV­SLI­DTS­SFQ­NQL­DQM­PRR­**YLA­GNH­EQE­FLR­**YQQ­EGS­EEE­ENE­GGN­IFS­GFK­R**DF**­**LED­ALN­VNR­R**IV**­**NKL­QGR­**NED­EEK­GAI­VK**V**­**KGG­LSI­TTP­PEK­EPR­QKR­GSR­QEE­DED­EDE­KRQ­PHR­HSR­QDE­DED­EKR­QPH­HHS­RGG­SKS­QRD­NGF­EET­ICT­ARL­HQN­IGS­SSS­PDI­YNP­QAG­RIK­TVT­SFD­LQA­LRF­LKL­SAE­FGS­LHK­NAM­FVP­HYN­LNA­NSI­LYA­LKG­RAR­LLY­ALN­CKG­NSV­FDG­ELE­AGR­ALI­VPQ­NFA­IAA­KSL­SDR­FSY­VAF­KTN­DRA­LIN­VCQ­KKL­LQL­LSI­WKE­MRP­GSS­SST­APF­HFL­FHP­AVT­QTT­KQQ­LDL­VPN­QYE	*Legumin*, alpha and beta, subunit precursor of *Cicer arietinum*	**56216**	**6.20**	**7**

6	ETT­AFN­TTS­RIG­NWS­SAI­SPP­LQT­CGS­FKC­QLP­TRR­**GVIV AD­LR**N**­**SNF­RWR­K**AT**­**TTS­RGN­VAA­EAV­KIP­TSV­PVR­VAR­EL AQAGYR**YLDVRT	*Arabidopsis thaliana*	**10249**	**11.14**	**45**
